# An elevated level of the mRNA exporter Mex67-Mtr2 in nuclear mRNPs impairs nuclear mRNA export

**DOI:** 10.1093/nar/gkag025

**Published:** 2026-01-23

**Authors:** Nataliia Stefanyshena, Katja Sträßer

**Affiliations:** Institute of Biochemistry, FB08, Justus Liebig University Giessen, Heinrich-Buff-Ring 17, 35392 Giessen, Germany; Institute of Biochemistry, FB08, Justus Liebig University Giessen, Heinrich-Buff-Ring 17, 35392 Giessen, Germany; Cardio-Pulmonary Institute (CPI), EXC 2026, 35392 Giessen, Germany

## Abstract

In eukaryotes, nuclear messenger RNA (mRNA) export is a crucial step in gene expression, mediated by the conserved mRNA exporter Mex67-Mtr2 in *Saccharomyces cerevisiae* and NXF1-NXT1 in humans. Mex67-Mtr2 is recruited to the mRNA by the adaptors Hpr1, Nab2, Yra1, and Npl3, which play important yet incompletely understood roles in this process. Here, we uncover that, counterintuitively, an excess of Mex67 in nuclear messenger ribonucleoprotein particles (mRNPs) impairs nuclear mRNA export. Cells lacking Hpr1, which exhibit a nuclear mRNA export defect, show elevated levels of Nab2, Yra1, and Mex67 in nuclear mRNPs. Remarkably, overexpression of either Nab2 or Yra1 in *Δhpr1* cells suppresses this export defect and simultaneously decreases the Mex67 level in nuclear mRNPs to those of wild-type cells. Importantly, a nuclear mRNA export defect is not inherently associated with an elevated Mex67 level in nuclear mRNPs, indicating that the increased Mex67 level in nuclear mRNPs of *Δhpr1* cells is likely the cause rather than the consequence of the nuclear mRNA export defect. Thus, the precise regulation of the Mex67-Mtr2 level in nuclear mRNPs is essential for efficient nuclear mRNA export.

## Graphical Abstract

**Graphical Abstract:**
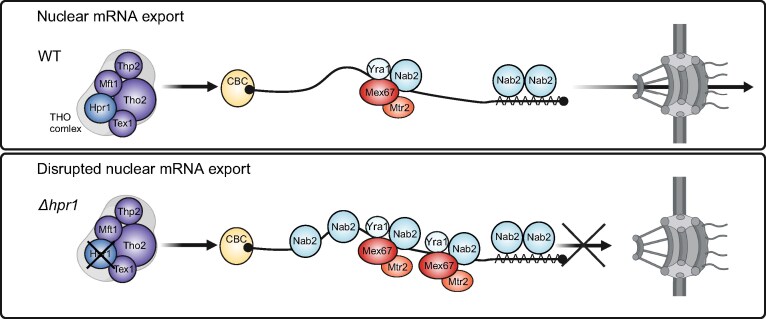

## Introduction

Gene expression is an essential process for every cell and organism. In eukaryotic cells, RNA polymerase II (RNAPII) first transcribes a protein-coding gene into a messenger RNA (mRNA). Largely already co-transcriptionally, the mRNA is processed by addition of a cap structure to its 5′ end, splicing of the introns, and addition of a poly(A) tail after cleavage at its 3′ end [1, 2]. Importantly, the mRNA is also packaged into a messenger ribonucleoprotein particle (mRNP) by nuclear RNA-binding proteins (RBPs), so-called mRNP components [1–3]. These processes, as well as the involved proteins, are well-conserved [3, 4]. Most likely, all nuclear mRNP components, which have multiple functions along the gene expression pathway, are known in the model organism *Saccharomyces cerevisiae* [5].

The TREX complex couples transcription elongation to nuclear mRNA export, most likely by binding to the nascent mRNA and facilitating mRNP assembly [4, 6]. It consists of the pentameric THO complex, which is composed of Tho2, Hpr1, Mft1, Thp2, and Tex1; the SR-like proteins Gbp2 and Hrb1; and the mRNA export factors Sub2 and Yra1 [6]. The cap binding complex (CBC), consisting of Cbc1 and Cbc2, binds to the 5′ end of the mRNA and promotes nuclear mRNA export by recruiting Yra1 and Npl3 to chromatin in a THO- and Sub2-independent manner [7]. Tho1 is a conserved nuclear mRNP component proposed to function complementarily to Sub2, as overexpression of either suppresses *Δhpr1* defects [8]. It is recruited to transcribed genes in a THO- and RNA-dependent manner [8]. CIP29, its homolog in humans, interacts with TREX and UAP56/DDX39, the human homolog of Sub2, and mutation of MOS11, the Tho1 homolog in plants, causes a nuclear mRNA export defect [9, 10]. Thus, the protein Tho1/CIP29/MOS11 is critical for mRNP assembly and nuclear mRNA export, though its exact functions remain unclear. Several SR- and SR-like mRNP components are involved in packaging mRNAs into mRNPs [11]. In *S. cerevisiae*, the SR-like protein Npl3 plays roles in transcription, splicing, 3′ end processing, and mRNA export [12–14]. The nuclear poly(A)-binding protein Nab2 functions in poly(A) tail length control, nuclear mRNP assembly, and export [15]. It is recruited to the site of transcription by binding to the RNA [15]. Similarly, in human cells, several nuclear mRNP components are SR- and SR-like proteins and are important for nuclear mRNA export [11]. The THSC complex (TREX2), consisting of Thp1, Sac3, Sus1, Cdc31, and Sem1, is also essential for nuclear mRNA export [16]. It interacts with Mex67-Mtr2/NXF1-NXT1 and the nuclear pore complex (NPC) [16]. However, the exact functions and mechanisms of THSC remain unclear. Finally, the mRNP is exported out of the nucleus by the conserved mRNA exporter Mex67-Mtr2 in *S. cerevisiae*/NXF1-NXT1 in human cells. Thus, Mex67-Mtr2/NXF1-NXT1 is essential for nuclear mRNA export [17]. As the intrinsic affinity of Mex67-Mtr2 for RNA is very low, four export adaptor proteins, Hpr1, Nab2, Yra1, and Npl3, recruit the mRNA exporter to the mRNA in *S. cerevisiae* [18–21]. In mammalian cells, TREX (via its components ALYREF and THOC5), CHTOP, UIF, several SR proteins, and ZC3H3 recruit NXF1-NXT1 to the mRNA [22]. Mex67-Mtr2/NXF1-NXT1 also binds to nuclear pore proteins and exports the mRNP out of the nucleus. On the cytoplasmic side of the NPCs, several mRNP components, including Mex67-Mtr2, are dissociated from the mRNA, rendering nuclear mRNA export unidirectional [23, 24].

Nuclear mRNP assembly and export are tightly regulated, particularly under stress, likely through the controlled recruitment of Mex67-Mtr2, a process that remains poorly understood [25–27]. Despite its importance, it is still unclear how an mRNA and its associated nuclear mRNP components assemble into an mRNP and what the precise functions of the individual mRNP components are. In particular, it remains to be elucidated why at least four different adaptor proteins exist in *S. cerevisiae*, how they cooperate, and how they functionally compensate for one another.

Here, we show that not only a reduced but also an increased level of the mRNA exporter Mex67-Mtr2 in nuclear mRNPs causes a nuclear mRNA export defect. Deletion of *HPR1*, which encodes one of the adaptor proteins, leads to a nuclear mRNA export defect and higher levels of Nab2 and Yra1, two other adaptors, and of Mex67 in nuclear mRNPs. Strikingly, overexpression of either Nab2 or Yra1 in *Δhpr1* cells suppresses the mRNA export defect and at the same time decreases the Mex67 level in nuclear mRNPs to those observed in wild-type cells. Importantly, we show that the increased Mex67 level in nuclear mRNPs of *Δhpr1* cells is the cause, rather than the consequence, of the nuclear mRNA export defect. These findings indicate that maintaining an optimal Mex67 level in nuclear mRNPs is essential for efficient nuclear mRNA export.

## Materials and methods

### Strains, plasmids, and primers

Yeast strains, plasmids, and primers are listed in Supplementary Tables S1-S3, respectively.

### Purification of nuclear mRNPs

Nuclear mRNPs were isolated by purification of endogenously TAP-tagged Cbc2, a subunit of CBC, as described in [12] with some modifications. Briefly, 2 l of yeast culture were harvested at OD_600_ 3.5 and lysed by cryogenic grinding (6870D Freezer/Mill, SPEX Sample Prep). The grindates were mixed with 10 ml lysis buffer (50 mM HEPES–KOH, pH 7.5, 100 mM KCl, 1.5 mM MgCl_2_, 0.15% NP-40, 1 mM DTT, 1× protease inhibitor), pre-cleared by centrifugation at 3500 × *g* for 12 min, and spun at 165 000 × *g* for 1 h at 4°C. The supernatants were incubated with 500 μl of IgG Sepharose 6 Fast Flow beads (GE Healthcare) for 1.5 h at 4°C. The beads were washed with 15 ml wash buffer (50 mM HEPES–KOH, pH 7.5, 200 mM KCl, 1.5 mM MgCl_2_, 0.15% NP-40, 0.5 mM DTT) and incubated with TEV protease for 1 h at 16°C to elute bound proteins. Proteins in lysate and eluate were analyzed by western blotting using the respective primary antibodies against the protein of interest, followed by incubation with a horseradish peroxidase-coupled secondary antibody. Chemiluminescent signals were detected with CheLuminate-HRP ECL solution (Applichem) using a ChemoCam Imager (Intas). Protein intensities were quantified using ImageJ software.

### RNA immunoprecipitation

RNA immunoprecipitation (RIP) was performed as described in [12]. 400 ml of cells expressing the endogenously TAP-tagged protein of interest were harvested at OD_600_ 0.8, resuspended in 1 ml RNA-IP buffer (25 mM Tris–HCl, pH 7.5, 150 mM NaCl, 2 mM MgCl_2_, 0.2% Triton X-100, 0.5 mM DTT, 2× protease inhibitor), and lysed with glass beads using a FastPrep-24 5G device (3× for 20 s at 6 m/s). Cell debris was cleared by centrifugation for 15 min at 16 000 × *g* at 4°C. 900 μl of the lysate were incubated with 660 units of DNaseI for 30 min on ice (Input). 35 μl of IgG-coupled Dynabeads (Thermo Scientific) were added to the lysate and incubated for 3 h at 4°C on a rotating wheel. The beads were washed 8× with 1 ml RNA-IP buffer. 1 ml of TRIzol was added to the beads for RNA extraction (IP). Isolated RNA from input and IP samples was reverse-transcribed for subsequent quantitative PCR (qPCR) analysis using the Applied Biosystems StepOnePlus cycler. Standard curves were used to determine qPCR efficiency (E). mRNA enrichment was calculated relative to the untagged negative control (nc) according to the formula *[E^(Ct IP – Ct Input)nc]/[E^(Ct IP- Ct Input)gene]*.

### Chromatin immunoprecipitation

Chromatin immunoprecipitation (ChIP) was performed as described in [28]. 100 ml of cell culture were grown to OD_600_ 0.8 and cross-linked with 1% formaldehyde for 20 min. Cells were washed, resuspended in 800 μl low-salt buffer (50 mM HEPES–KOH, pH 7.5, 150 mM NaCl, 1 mM EDTA, 1% Triton X-100, 0.1% SDS, 0.1% sodium deoxycholate, 1× protease inhibitor), and lysed with glass beads in a FastPrep-24 5G device (2 × 45 s at 6 m/s). Chromatin was fragmented to 200–250 bp by sonication with a Bioruptor UCD-200 (Diagenode) 3× for 15 min (30 s ON /30 s OFF; “HIGH” mode). Lysates were cleared by centrifugation for 15 min at 16 000 × *g* at 4°C (input). To immunoprecipitate a TAP-tagged protein, the lysate was incubated with 15 μl IgG-coupled Dynabeads M-280 (Thermo Scientific) for 2.5 h at room temperature. For RNAPII ChIP, the lysate was incubated with 4 μl of 8WG16 antibody (BioLegend) for 1.5 h, followed by the addition of 15 μl Dynabeads Protein G (Thermo Scientific) for 1 h. Beads were washed with 800 μl of buffer [2× low-salt buffer, 3× high-salt buffer (500 mM NaCl), 3× TLEND, and 2× with 1× TE, pH 8.0], and DNA–protein complexes were eluted (IP). Input and IP samples were incubated with proteinase K for 2 h at 37°C, followed by reversal of the crosslinks for 14 h at 65°C. DNA was purified with the PCR NucleoSpin^®^ Gel and PCR Clean-up kit (Macherey-Nagel) and used for qPCR analysis. Standard curves were used to determine the qPCR efficiency (E). As a negative control, a non-transcribed region (NTR) on chromosome V (174 131–174 200) was used. Protein occupancy was calculated as the enrichment of the sequence for the gene of interest relative to the NTR using the formula *[E^(Ct IP – Ct Input)NTR]/[E^(Ct IP – Ct Input)Gene]*.

### Dot spot assay

Yeast cells were resuspended in 2 ml water. The cell suspensions were diluted to the same OD_600_ of 0.09 for each strain (1st spot). 5 µl of 10-fold serial dilutions were spotted on selective media plates. Cells were grown at 25°C, 30°C, and 37°C for 2 days and at 16°C for up to 6 days before imaging.

### Fluorescence *in situ* hybridization

The cellular localization of poly(A)^+^ RNA was determined by fluorescence *in situ* hybridization (FISH) performed as described previously [12] with some modifications. Cells were grown to an OD_600_ 0.8 at 30°C or shifted to 37°C for 1 h before crosslinking with 4% formaldehyde for 90 min. Washed cells were incubated with 100 μg of 100T zymolase for 30 min at 30°C to digest the cell walls. Obtained spheroplasts were applied to polylysine-coated coverslips. The adherent cells were incubated with 200 μl of 2× SSC buffer for 10 min, followed by incubation with 12 μl of prehybridization buffer (50% formamide, 10% dextran sulphate, 125 μg/ml of *Escherichia coli* tRNA, 500 μg/ml herring sperm DNA, 4× SSC, 0.02% polyvinyl pyrrolidone, 0.02% bovine serum albumin, 0.02% Ficoll-40) for 1 h at 37°C in a humid chamber. 0.75 μl of 1 pmol/μl oligo(dT)_50_-Cy3 probe was added and incubated at 37°C overnight in a humid chamber. After hybridization, coverslips with cells were washed with 3 ml of 0.5× SSC for 30 min on a rocking shaker, air-dried, and mounted with ROTI^®^ Mount FluorCare DAPI (Carl Roth) on microscope slides. Cells were imaged using a Deltavision Ultra High-Resolution Microscope (Cytiva).

### Statistical analyses

All experiments were performed with at least three independent biological replicates. Data are presented as mean ± standard deviation (error bars). Asterisks indicate statistical significance (Student’s *t*-test; *P ≤ .05; **P ≤ .01; ***P ≤ .001).

## Results

### Loss of TREX causes a nuclear mRNA export defect and higher levels of the mRNP components Nab2 and Yra1 and the mRNA exporter Mex67 in nuclear mRNPs

For nuclear mRNA export, adaptor proteins recruit the conserved mRNA exporter Mex67-Mtr2/NXF1-NXT1 to the nuclear mRNA [11, 17, 29]. In *S. cerevisiae*, four such adaptors have been identified: Hpr1, Nab2, Yra1, and Npl3 [18–21]. The existence of several adaptor proteins as well as experimental evidence suggests different export pathways for different classes of mRNAs. However, the molecular consequences of the lack of one of these potentially redundant export adaptors are largely unknown, especially in terms of nuclear mRNP biogenesis. Thus, we assessed the effects of lack of Hpr1, one of the four adaptors. To do so, the non-essential gene coding for Hpr1 was either deleted or Hpr1 depleted using the auxin-inducible degron system (Supplementary Fig. S1A and B). Loss of Hpr1 by deletion or depletion causes a growth defect (Supplementary Fig. S1C and [30]) and a concomitant nuclear mRNA export defect (Supplementary Fig. S1D and [6, 31]). Interestingly, in the absence of Hpr1, Thp2 still interacts with Mft1 but no longer with Tho2 (Supplementary Fig. S1E). This is consistent with the recently published cryo-EM-based structural model of the *S. cerevisiae* THO complex (Supplementary Fig. S1F and [32]). Thus, lack of Hpr1 leads to disassembly of the THO complex, and the effects observed for cells lacking Hpr1 are probably due to the absence of a functional THO complex.

**Figure 1.**
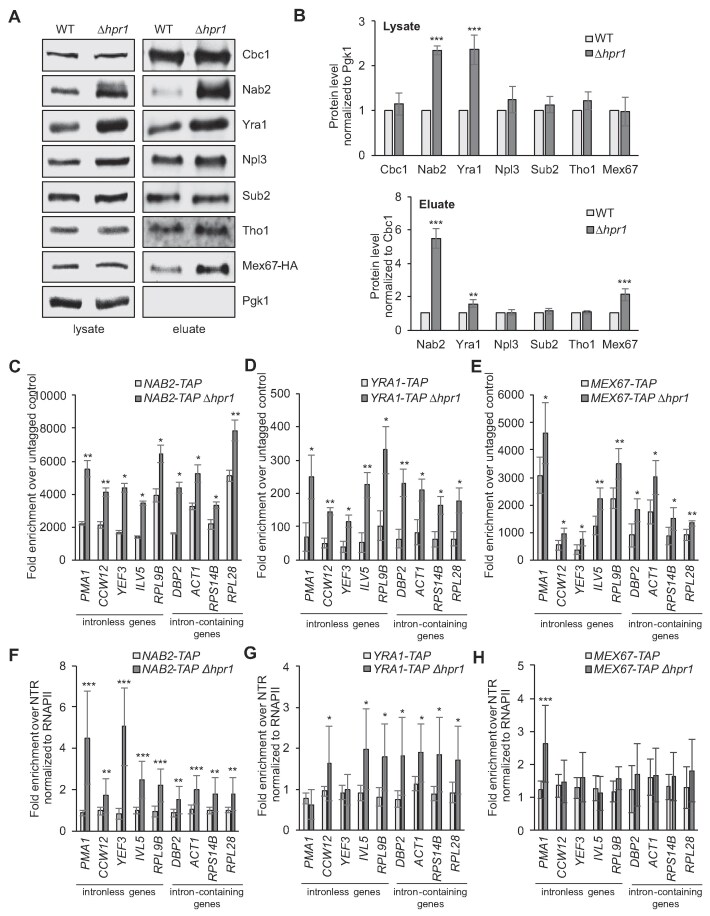
Loss of Hpr1 leads to increased Nab2, Yra1, and Mex67 levels in nuclear mRNPs. (**A**) Representative western blots of lysates and TEV eluates after Cbc2-TAP purification from wild-type (WT) and *Δhpr1* cells, using antibodies against the indicated RBPs. (**B**) Quantification of the protein levels in lysates (upper panel) and TEV eluates (lower panel) of six independent experiments, normalized to Pgk1 or Cbc1 levels, respectively. Values for WT cells were set to 1. (**C - E**) RNA binding of Nab2 (C), Yra1 (D), and Mex67 (E) increases in *∆hpr1* cells. TAP-tagged Nab2, Yra1, or Mex67 were immunoprecipitated, and the amount of co-immunoprecipitated RNA was determined by RT-qPCR for representative abundant intronless and intron-containing transcripts. mRNA levels were quantified as the enrichment over the mRNA amounts in cells expressing untagged proteins. (**F - H**) The occupancy of Nab2 (F) and Yra1 (G) at transcribed genes is increased in *∆hpr1* cells, while the occupancy of Mex67 (H) remains unchanged. ChIP analysis was used to assess the occupancy of TAP-tagged proteins at genes. Co-immunoprecipitated DNA was quantified by qPCR for the same intronless and intron-containing genes as in panels (C - E). The occupancy was calculated as the enrichment of protein at the genes relative to its presence at a non-transcribed region (NTR, 174 131–174 200 on chr. V), normalized to RNAPII occupancy, and set to 1 for WT cells. Data represent the mean ± SD of at least three independent experiments; *P < .05; **P < .01; ***P < .001.

To assess potential changes in nuclear mRNP composition that might result from loss of Hpr1 and, thus, a functional THO complex, we purified nuclear mRNPs by native pulldown of endogenous TAP-tagged Cbc2, the small subunit of CBC. Interestingly, while the level of Npl3, the fourth adaptor, is neither increased in the *HPR1* deletion strain nor after Hpr1 depletion (Fig. 1A and B and Supplementary Fig. S1G and H), the absence of Hpr1 causes higher levels of Nab2 and Yra1, two of the three other export adaptors, in whole cell lysates and in nuclear mRNPs (Fig. 1A and B).

This is consistent with our observation that Nab2 levels in nuclear mRNPs increase after 1 h of Hpr1 depletion, whereas both Nab2 and Yra1 levels are elevated after 3 h of depletion (Supplementary Fig. S1G and H). However, quite unexpectedly, the level of the mRNA exporter Mex67 is also increased in nuclear mRNPs in *Δhpr1* cells compared to WT cells (Fig. 1A and B), despite the presence of an mRNA export defect. The Mex67 level in nuclear mRNPs also increases after 3 h of Hpr1 depletion (Supplementary Fig. S1G and H). Notably, the extent of the increase differs among Nab2, Yra1, and Mex67. The particularly pronounced increase in the Nab2 level may reflect its ability to dimerize and/or its binding to the poly(A) tail or to A-rich regions within mRNAs that are less compacted in the absence of TREX. In summary, the Mex67 level in nuclear mRNPs increases in *Δhpr1* cells, even though nuclear mRNA export is defective in these cells.

The increased levels of Nab2, Yra1, and Mex67 in nuclear mRNPs of *Δhpr1* cells suggest that these proteins bind better to mRNA. To assess this, we performed RIP experiments by purification of genomically TAP-tagged proteins from whole cell extracts of WT and *Δhpr1* cells (Fig. 1C–E). Coimmunoprecipitated RNA was analyzed by RT and qPCR for five representative abundant intronless transcripts (*PMA1, CCW12, YEF3, ILV5*, and *RPL9B*) and four intron-containing transcripts (*DBP2, ACT1, RPS14B*, and *RPL28*). Indeed, the three proteins bind higher amounts of these mRNAs in the *Δhpr1* compared to the WT strain (Fig. 1C–E). Thus, nuclear mRNPs contain more Nab2, Yra1, and Mex67 in *Δhpr1* cells.

Nuclear mRNP components are already recruited to the transcribed gene. To determine whether the presence of Nab2, Yra1, and Mex67 is increased at genes in *Δhpr1* cells, we assessed their occupancy by ChIP (Fig. 1F–H). As the occupancy of nuclear RBPs depends on the presence of RNA and thus on transcription, we first assessed the occupancy of RNAPII at genes encoding the same nine representative transcripts assayed by RIP. RNAPII occupancy decreases in the *Δhpr1* strain (Supplementary Fig. S2). Accordingly, the occupancy of all RBPs was normalized to the one of the RNAPII. Interestingly, the occupancy of Nab2 and Yra1, but not of the mRNA exporter Mex67, increases in *Δhpr1* cells (Fig. 1F–H). Taken together, lack of the THO complex leads to a higher occupancy of the adaptor proteins Nab2 and Yra1 at transcribed genes and to higher levels of these proteins in nuclear mRNPs. Likely, these two adaptor proteins then increase the Mex67 level in nuclear mRNPs.

### Overexpression of Nab2 or Yra1 suppresses the growth and the nuclear mRNA export defect of *Δhpr1* cells

**Figure 2.**
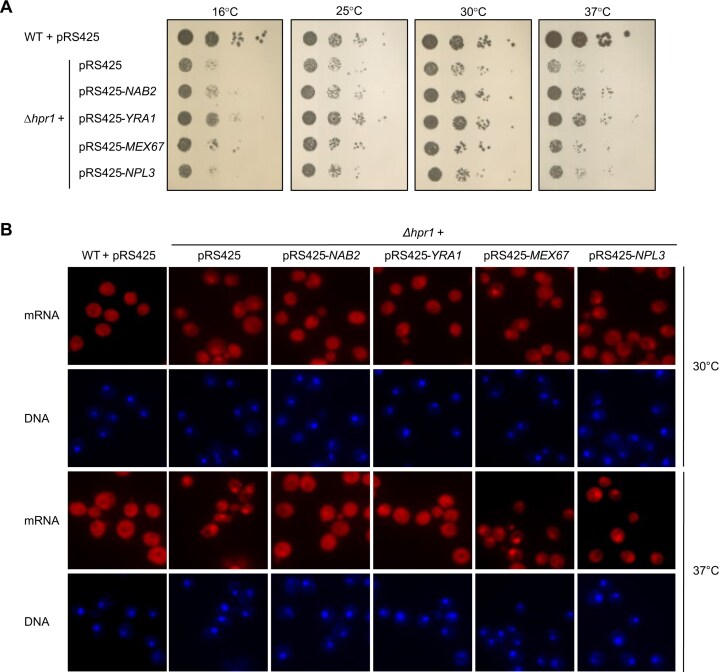
Overexpression of Nab2 or Yra1 suppresses the growth and nuclear mRNA export defects of *∆hpr1* cells. (**A**) The growth defect of *∆hpr1* cells is alleviated by overexpression of Nab2 or Yra1 but not by overexpression of Mex67 or Npl3. Ten-fold serial dilutions of WT or *∆hpr1* cells or *∆hpr1* cells overexpressing Nab2, Yra1, Mex67, or Npl3 were spotted onto SDC (-leu) plates and grown at the indicated temperatures. (**B**) The nuclear mRNA export defect of *Δhpr1* cells is suppressed by overexpression of Nab2 or Yra1 but not by overexpression of Mex67 or Npl3. Nuclear mRNA export was assessed by FISH of the indicated cells grown at 30°C or after a 1 h shift to 37°C. mRNA was detected using an oligo(dT)_50_ probe conjugated to the fluorescent dye Cy3. DNA was stained with DAPI. Representative images from three independent experiments are shown.

As a lack of Hpr1 leads to higher levels of Nab2, Yra1, and Mex67 in nuclear mRNPs, we hypothesized that overexpression of these proteins alleviates the growth and the nuclear mRNA export defect of *Δhpr1* cells. Overexpression of Npl3, whose level is not increased in nuclear mRNPs of *Δhpr1* cells, served as a potential negative control. As expected, overexpression of Npl3 in *Δhpr1* cells neither suppresses the growth nor the nuclear mRNA export defect of *Δhpr1* cells (Fig. 2A and B). Interestingly, overexpression of Nab2 or Yra1 partially suppresses the growth defect as well as the nuclear mRNA export defect of *Δhpr1* cells, whereas overexpression of Mex67 does not suppress either defect (Fig. 2A and B).

This is surprising, as the Mex67 level in nuclear mRNPs also increases in *Δhpr1* cells and as Mex67-Mtr2 is the mRNA exporter. Thus, an increased total Mex67-Mtr2 level might be insufficient, because Mex67-Mtr2 might need to be recruited to the nuclear mRNA, e.g. by Nab2 and/or Yra1. Taken together, overexpression of one of the two mRNA export adaptors Nab2 or Yra1 suppresses both defects, suggesting that the growth defect of *Δhpr1* cells is at least partially caused by their nuclear mRNA export defect.

### Overexpression of Nab2 or Yra1 in *Δhpr1* cells increases Nab2 levels even further and decreases Mex67 levels in nuclear mRNPs

**Figure 3.**
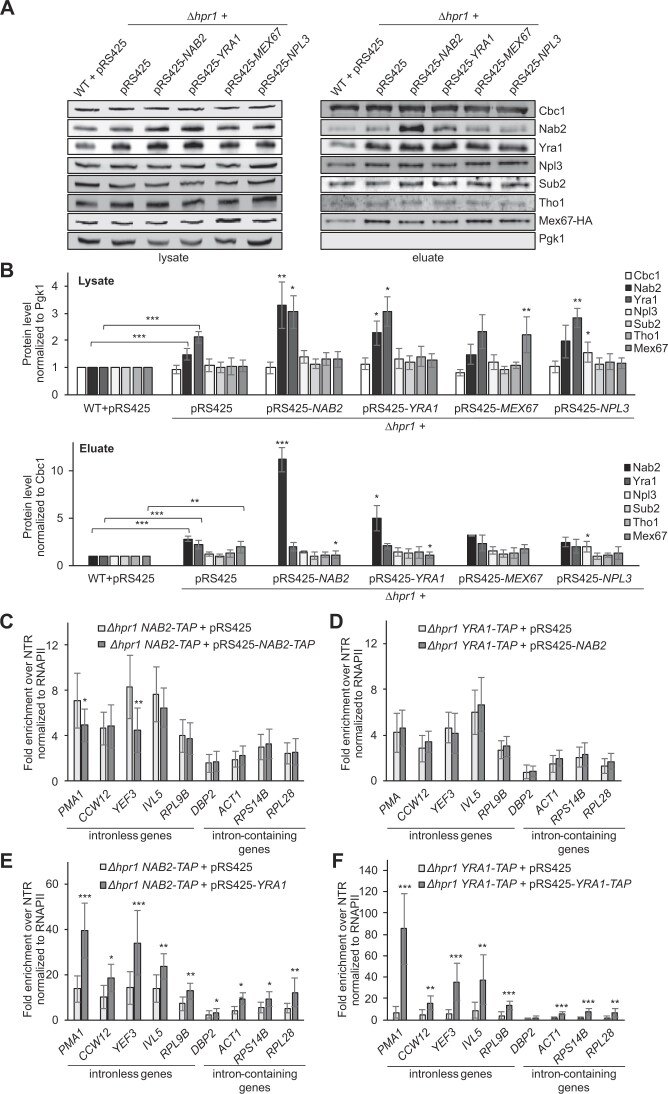
Overexpression of Nab2 or Yra1 in *Δhpr1* cells leads to a further increase of the Nab2 level and a decrease in the Mex67 level in nuclear mRNPs. (**A**) Representative western blots of lysates and TEV eluates of nuclear mRNPs obtained by Cbc2-TAP purification, using antibodies against indicated RBPs. (**B**) Quantification of protein levels in lysates and TEV eluates of six independent experiments were normalized to the signal of Pgk1 or Cbc1, respectively. Protein levels for WT cells were set to 1. Asterisks with brackets represent the comparison between WT and *Δhpr1* cells, and asterisks without brackets indicate the comparison to *Δhpr1* cells. (**C and D**) The occupancy of Nab2 (C) or Yra1 (D) at transcribed genes does not change upon Nab2 overexpression in *∆hpr1* cells. (**E and F**) The occupancy of Nab2 (E) and Yra1 (F) increases upon Yra1 overexpression in *∆hpr1* cells. Protein occupancy was assessed by ChIP as in Fig. 1F–H and normalized to the occupancy of RNAPII in the respective strains. Data represent the mean ± SD of at least three independent experiments; *P < .05; **P < .01; ***P < .001.

To unravel the molecular basis for the suppression of the nuclear mRNA export defect in *Δhpr1* cells by overexpression of Nab2 or Yra1, we determined the composition of nuclear mRNPs of these cells (Fig. 3A and B). The total protein amount of both Nab2 and Yra1 in *Δhpr1* cells increases further by overexpression of either Nab2 or Yra1 (Fig. 3A and B). Thus, the total amounts of these two proteins in cells are interdependent. However, in nuclear mRNPs, only the already elevated Nab2 level increases further upon overexpression of either Nab2 or Yra1 in *Δhpr1* cells, whereas the Yra1 level is unaffected (Fig. 3A and B). Surprisingly, the Mex67 level in nuclear mRNPs decreases to WT levels in *Δhpr1* cells when either Nab2 or Yra1 is overexpressed (Fig. 3A and B). In contrast, the levels of all assessed RBPs remain unchanged when Mex67 or Npl3 is overexpressed (Fig. 3A and B). Thus, when the nuclear mRNA export defect caused by the absence of Hpr1 is suppressed, the Nab2 level in nuclear mRNPs is even higher than in *Δhpr1* cells, and the Mex67 level decreases to WT. This suggests that a wild-type level of Mex67 is necessary for efficient nuclear mRNA export.

To determine whether the increased Nab2 level in nuclear mRNPs of *Δhpr1* cells by overexpression of Nab2 or Yra1 is already present at transcribed genes, we assessed the occupancy of Nab2 and Yra1 by ChIP (Fig. 3C–F). When Nab2 is overexpressed in *Δhpr1* cells, the occupancy of Nab2 or Yra1 does not change (Fig. 3C and D). Thus, “additional” Nab2 is most likely incorporated into nuclear mRNPs after transcription. In contrast, overexpression of Yra1 in *Δhpr1* cells leads to a higher occupancy of Nab2 as well as of Yra1 (Fig. 3E and F). This increased occupancy correlates with higher levels of Nab2 but not of Yra1 in nuclear mRNPs (Fig. 3A and B). Thus, Nab2 and Yra1 act in an overlapping yet different manner: Nab2 overexpression does not change Nab2 or Yra1 occupancy but results in a higher Nab2 level in nuclear mRNPs. In contrast, overexpression of Yra1 increases the occupancy of Yra1 and Nab2 but yields only elevated levels of Nab2 in nuclear mRNPs, suggesting that Yra1 either recruits Nab2 or stabilizes its interactions for its consecutive loading onto mRNPs. This is consistent with the finding that Yra1 is not absolutely required for nuclear mRNA export and rather acts as a cofactor favoring the interaction between Nab2 and Mex67 [20]. In summary, higher levels of Nab2 or Yra1 suppress the mRNA export defect of *Δhpr1* cells and reduce the levels of Mex67 in nuclear mRNPs to WT levels.

### Nuclear pore complex mutants with a nuclear mRNA export defect have higher levels of Nab2, Yra1, and Mex67 in nuclear mRNPs but are not suppressed by overexpression of Nab2 or Yra1

**Figure 4.**
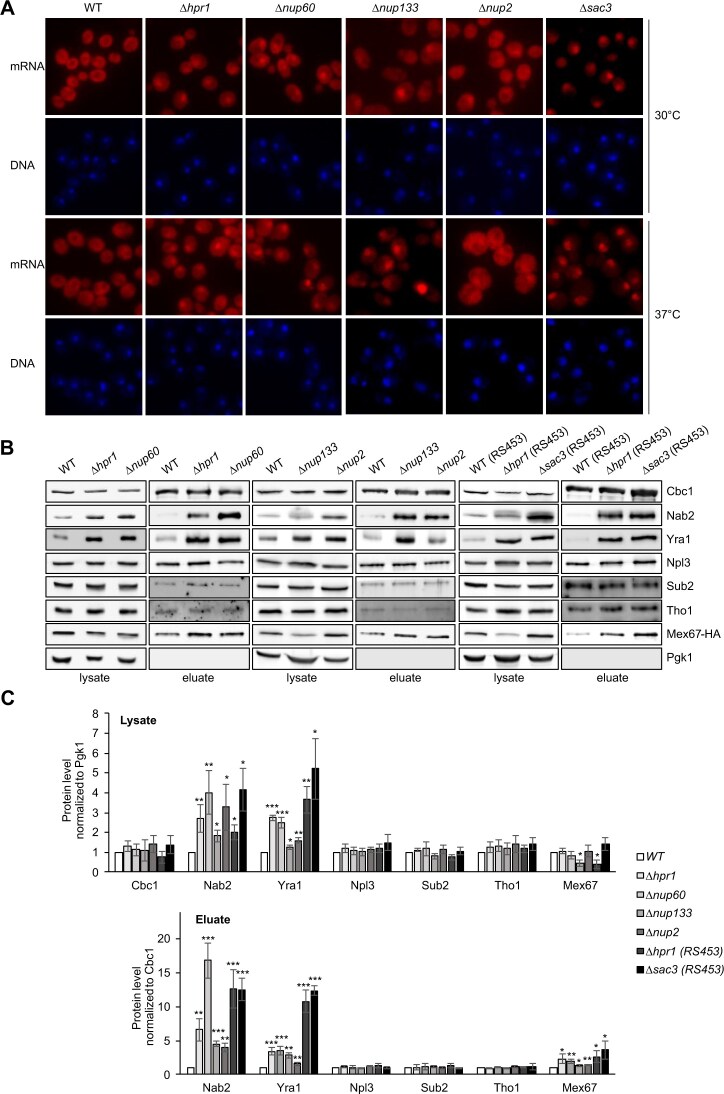
Deletion of *NUP60, NUP133, NUP2*, or *SAC3* results in an increase of Nab2, Yra1, and Mex67 levels in nuclear mRNPs. (**A**) Nuclear mRNA export is impaired in *Δhpr1, Δnup60, Δnup133, Δnup2*, and *Δsac3* cells. Poly(A)^+^ RNA was visualized by FISH at 30°C or after 1 h shift to 37°C with a Cy3-conjugated oligo(dT)_50_ probe. DNA was stained with DAPI. (**B**) Representative western blots of lysates and TEV eluates after Cbc2-TAP purification from WT, *Δnup60, Δnup133, Δnup2*, and *Δsac3* cells, using antibodies against the indicated RBPs. (**C**) Quantification of protein levels in lysates and TEV eluates, normalized to Pgk1 or Cbc1, respectively. Values for WT cells were set to 1. Data represent the mean ± SD of three independent experiments; *P < .05; **P < .01; ***P < .001. Unless otherwise indicated, all used strains have a W303 background. As deletion of *SAC3* causes a stronger nuclear mRNA export defect in the RS453 than in the W303 background, the *Δsac3* strain, and for comparison the WT and *∆hpr1* strains, have an RS453 background.

To examine whether the increased Nab2 and Yra1 level in nuclear mRNPs is a general phenomenon of nuclear mRNA export mutants, we analyzed strains with deletion of *NUP60, NUP133*, or *NUP2* coding for nuclear pore proteins or of *SAC3* coding for a THSC/TREX2 component, all of which exhibit a nuclear mRNA export defect (Fig. 4A and [16]). Interestingly, the Nab2, Yra1, and Mex67 levels in nuclear mRNPs in all four mutants are increased to a similar extent as in *Δhpr1* cells (Fig. 4B and C). In contrast, overexpression of Yra1 or Nab2 does not suppress the nuclear mRNA export defect of *Δnup60, Δnup133, Δnup2*, and *Δsac3* cells (Supplementary Fig. S3A–D). To determine the potential reason for this lack of suppression, we analyzed the composition of nuclear mRNPs for one of these strains, *Δnup60*. As in the *Δhpr1* mutant, overexpression of either Nab2 or Yra1 leads to higher levels of Nab2 in nuclear mRNPs of the *Δnup60* mutant (Supplementary Fig. S3E and F). In contrast to *Δhpr1* cells, not only the Nab2 level but also the Yra1 level in nuclear mRNPs increases due to overexpression of Nab2 or Yra1 (Supplementary Fig. S4D and E). Importantly, the Mex67 level in nuclear mRNPs is still elevated compared to WT in *Δnup60* cells with Nab2 or Yra1 overexpression (Supplementary Fig. S3E and F), concomitant with the persisting nuclear mRNA export defect. Thus, an mRNA export defect caused by mutation at a later stage of nuclear mRNA export, i.e. at the nuclear pore, apparently cannot be suppressed by overexpression of Nab2 or Yra1.

### Depletion of Sub2 causes a nuclear mRNA export defect but does not lead to an increased Mex67 level in nuclear mRNPs

**Figure 5.**
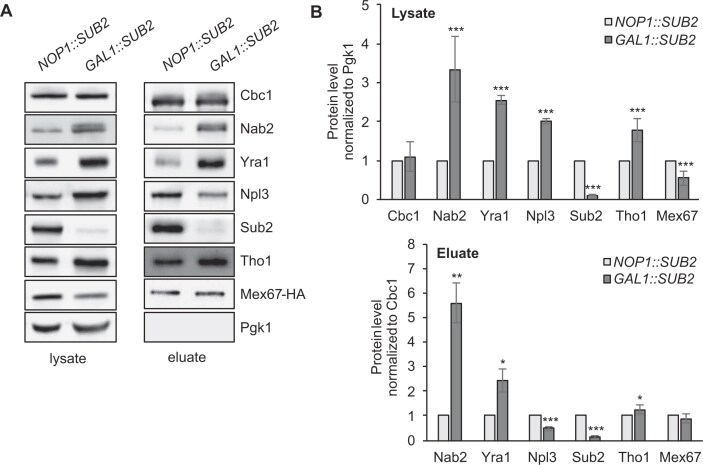
A nuclear mRNA export defect does not always cause an elevated Mex67 level in nuclear mRNPs. (**A**) Representative western blots of lysates and TEV eluates after Cbc2-TAP purification from cells expressing Sub2 (*pNOP1::SUB2*) or after depletion of Sub2 (*pGAL1::SUB2*), using antibodies against the indicated RBPs. Cells either expressing Sub2 from a constitutive promoter (*pNOP1::SUB2*) or from a galactose-inducible promoter (*pGAL1::SUB2*) were shifted from galactose- to glucose-containing medium for 20 h to deplete Sub2. (**B**) Quantification of protein levels of lysates and TEV eluates, normalized to Pgk1 or Cbc1, respectively. Values for WT cells were set to 1. Data represent the mean ± SD of three independent experiments; *P < .05; **P < .01; ***P < .001.

Interestingly, the level of the mRNA exporter Mex67 in nuclear mRNPs is increased in *Δhpr1* cells, which have an mRNA export defect, and is reduced to WT level in *Δhpr1* cells by overexpression of Nab2 or Yra1, which concomitantly represses the nuclear mRNA export defect. Thus, the increased Mex67 level in nuclear mRNPs could be either the cause or the consequence of the nuclear mRNA export defect. As depletion of the nuclear mRNP component Sub2 causes a nuclear mRNA export defect [33], we determined the composition of nuclear mRNPs after Sub2 depletion. As for the other mRNA export mutants, Nab2 and Yra1 levels in nuclear mRNPs increase (Fig. 5A and B). Furthermore, the Tho1 level increases, whereas the Npl3 level decreases in nuclear mRNPs (Fig. 5A and B). Importantly, the Mex67 level in nuclear mRNPs is unchanged (Fig. 5A and B). Thus, higher Nab2 and Yra1 levels in nuclear mRNPs do not necessarily result in an increase in the Mex67 level in nuclear mRNPs. Therefore, the elevated Mex67 level in nuclear mRNPs of cells lacking TREX, THSH, or nuclear pore proteins is likely not the result but rather the cause of defective nuclear mRNA export. In summary, not only the lack of the mRNA exporter Mex67-Mtr2 and thus its function, but also an increased level of Mex67 in nuclear mRNPs causes a defect in nuclear mRNA export.

## Discussion

Nuclear assembly of the mRNA into an mRNP is essential for its subsequent export. Nevertheless, how exactly the numerous mRNP components assemble onto the mRNA, as well as their stoichiometry and the architecture of a nuclear mRNP, remain unknown. Furthermore, many, if not all, mRNP components have multiple functions in gene expression. In *S. cerevisiae*, four adaptor proteins—Hpr1, Nab2, Yra1, and Npl3—recruit the mRNA exporter Mex67-Mtr2 to nuclear mRNAs. Yet, why multiple adaptors exist and how their functions differ is still unknown.

Unexpectedly, nuclear mRNPs of *Δhpr1* cells contain an excess of the mRNA exporter Mex67-Mtr2, coinciding with their nuclear mRNA export defect. How Mex67-Mtr2 binds to nuclear mRNAs remains unclear. In the nucleoplasm, Mex67-Mtr2 likely associates indirectly with mRNPs through protein-protein interactions with adaptors such as Nab2 and Yra1. In contrast, direct binding of Mex67-Mtr2 to mRNA—most likely the prerequisite for export—may occur at the nuclear side of the NPC [34], consistent with its removal from the mRNA by the helicase Dbp5 on the cytoplasmic side of the NPC. It is also unclear how the additional Mex67-Mtr2 associates with nuclear mRNPs in *Δhpr1* cells. Notably, in contrast to overexpression of Nab2 or Yra1, overexpression of Dbp5 fails to suppress the growth defect of *Δhpr1* cells (data not shown). This suggests that removal of excess Mex67-Mtr2 from nuclear mRNPs does not require Dbp5 activity and/or that these mRNPs fail to reach the cytoplasmic face of the NPC, where Dbp5 is activated.

A central function of the TREX complex is to facilitate nuclear mRNP compaction, thereby preventing aberrant interactions of the mRNA with other transcripts or with the template DNA strand, the latter leading to R-loop formation. mRNP compaction also protects the mRNA from unwanted interactions with other nuclear factors, including nucleases, and may limit excessive binding of Nab2, Yra1, and Mex67-Mtr2. Accordingly, loss of Hpr1 and, consequently, of the TREX complex will impair mRNP compaction, providing a plausible explanation for both the nuclear mRNA export defect and the increased association of Mex67-Mtr2 observed in *Δhpr1* cells. Notably, cells partially compensate for the absence of Hpr1 by increasing both the total Nab2 and Yra1 levels and their abundance in nuclear mRNPs. Nab2 and Yra1 may partially substitute for THO by binding to the mRNA and promoting compaction and/or preventing hybridization with the template strand or other transcripts [35, 36]. This compensatory response likely contributes to the viability of *Δhpr1* cells despite defects in mRNP assembly, compaction, and nuclear export. Consistent with this model, overexpression of Nab2 or Yra1 suppresses both the growth and the nuclear mRNA export defect of *Δhpr1* cells, possibly by further increasing mRNP compaction. In addition, increased Nab2 and Yra1 RNA binding could sterically limit access of Mex67-Mtr2, thereby preventing its excessive association with mRNA. An important, albeit technically challenging, next step will be to directly assess mRNP compaction in wild-type cells, *Δhpr1* cells, and *Δhpr1* cells overexpressing Nab2 or Yra1, e.g. by electron microscopy.

The fact that both Nab2 and Yra1 suppress the growth and nuclear mRNA export defect of *Δhpr1* cells suggests that Nab2 and Yra1 might have similar or overlapping functions. Consistently, Yra1 is dispensable for nuclear mRNA export in *Drosophila* and *Caenorhabditis elegans* [20, 37, 38]. Moreover, Yra1 might recruit Nab2 to be loaded onto the mRNP, because overexpression of Yra1 in *∆hpr1* cells leads to increased occupancy of both proteins, Yra1 and Nab2. Interestingly, the mRNA exporter Mex67-Mtr2 interacts directly with both adaptor proteins, and Yra1 enhances the interaction between Nab2 and Mex67, which is supported by the fact that Yra1 is not essential in cells overexpressing Nab2 or Mex67 [18, 20]. Taken together, in wild-type cells Nab2 and Yra1 might form a complex that recruits the mRNA export adaptor Mex67-Mtr2 to the nuclear mRNP.

In contrast, the adaptor protein Npl3 seems to be different from Nab2 and Yra1. Npl3 levels in nuclear mRNPs do not increase in *Δhpr1* cells, and Npl3 overexpression does not suppress the growth and nuclear mRNA export defects of *Δhpr1* cells. Consistently, Nab2 and Npl3 preferentially associate with a distinct set of mRNAs, as Npl3 largely associates with transcripts coding for ribosomal proteins and other highly expressed transcripts, while Nab2 associates with transcripts coding for proteins required for transcription [39]. Thus, either the mechanistic function or the subset of bound mRNAs might be different between Nab2/Yra1 and Npl3.

Interestingly, overexpression of Nab2 or Yra1 did not suppress other defects observed in *Δhpr1* cells, such as in transcription, transcriptional readthrough, poly(A) tail length, splicing, or pre-mRNA leakage (data not shown). As a proxy for transcription, RNAPII occupancy is reduced in *Δhpr1* cells (Supplementary Fig. S2). However, this transcriptional defect in *Δhpr1* cells is not suppressed by overexpression of Nab2 or Yra1, as RNAPII occupancy does not increase (data not shown). Furthermore, *Δhpr1* cells accumulate R-loop structures, composed of DNA–RNA hybrids and the displaced coding DNA strand, which contribute to genomic instability [40]. However, this higher amount of R-loops in *Δhpr1* cells is not suppressed by overexpression of Nab2 or Yra1 (data not shown). Likewise, although loss of Nab2 or Npl3 results in global transcriptional readthrough [13, 41], overexpression of Nab2 or Yra1 does not suppress readthrough at the tested exemplary genes in *Δhpr1* cells (data not shown). Nab2 is a nuclear poly(A) binding protein regulating poly(A) tail length, and a nuclear mRNA export defect often causes hyperadenylation [42, 43]. However, the poly(A) tails of bulk RNA as well as of single exemplary transcripts are of wild-type length in *Δhpr1* cells, and their length does not change due to overexpression of Nab2 or Yra1 (data not shown). Furthermore, splicing of a reporter transcript rather increases in *Δhpr1* cells, most likely reflecting their longer dwell time in the nucleus due to the mRNA export defect (data not shown). Finally, leakage of an intron-containing reporter transcript to the cytoplasm is increased in *Δhpr1* cells. This defect is also not suppressed by Nab2 or Yra1 overexpression (data not shown). Thus, overexpression of Nab2 or Yra1 in *Δhpr1* cells specifically suppresses the nuclear mRNA export defect and, most likely as a result, also their growth defect.

**Figure 6.**
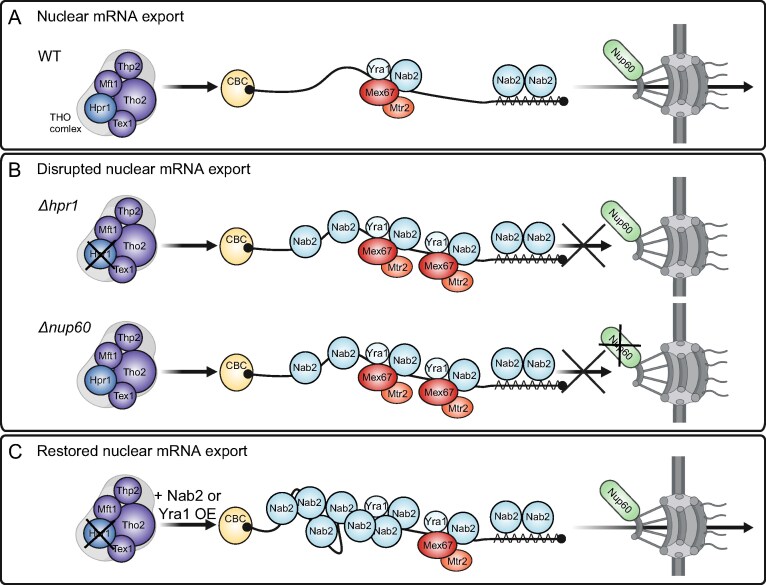
Nuclear mRNA export depends on a balanced level of the mRNA exporter Mex67-Mtr2 in nuclear mRNPs. (**A**) Nuclear mRNP assembly is one prerequisite for nuclear mRNA export. In a WT strain, the adaptor proteins Hpr1, Nab2, Yra1, and Npl3 recruit the mRNA exporter Mex67-Mtr2 to the nuclear mRNP for nuclear mRNA export. Npl3 and all other nuclear mRNP components are not shown for clarity. (**B**) Loss of Hpr1 and thus the THO complex causes increased levels of Nab2, Yra1, and Mex67 in nuclear mRNPs and a nuclear mRNA export defect. Disruption of mRNA export at the nuclear pore by deletion of *NUP60* or other nuclear pore components such as *NUP133, NUP2*, or *SAC3* results in similar changes in the composition of nuclear mRNPs and a nuclear mRNA export defect. (**C**) Overexpression of Nab2 or Yra1 in *∆hpr1* cells further increases the Nab2 level and decreases the Mex67 level to WT in nuclear mRNPs and restores nuclear mRNA export. Therefore, a wild-type level of Mex67 is required for efficient nuclear mRNA export.

The adaptor proteins Nab2 and Yra1 recruit the mRNA exporter Mex67-Mtr2 to the nuclear mRNP, enabling export of the mRNP through the nuclear pore complex (Fig. 6A). Loss of the THO complex leads to elevated levels of Nab2, Yra1, and Mex67 in nuclear mRNPs compared to wild-type cells and a nuclear mRNA export defect (Fig. 1 and Fig. 6B). Similar changes in mRNP composition occur when nuclear mRNA export is impaired due to the deletion of nuclear pore or THSC components (Fig. 6B).

Importantly, the increased Mex67 level in nuclear mRNPs is unlikely to be a mere consequence of the nuclear mRNA export defect, as depletion of the mRNP component Sub2 also causes a nuclear mRNA export defect but does not cause an increased Mex67 level in nuclear mRNPs (Fig. 5). Furthermore, overexpression of Nab2 or Yra1 not only suppresses the nuclear mRNA export defect of *Δhpr1* cells but also decreases the elevated Mex67 level in nuclear mRNPs to the one observed in wild-type cells (Figs 2B, 3A and B, and 6C). Consistently, overexpression of Mex67 in *Δhpr1* cells neither suppresses the mRNA export defect nor alters the composition of nuclear mRNPs (Figs 2B and 3A and B). The finding that suppression of the nuclear mRNA export defect coincides with a reduction in the Mex67 level in nuclear mRNPs is unexpected, as the current model suggests that Mex67-Mtr2 recruitment is essential for nuclear mRNA export. This contrasts with the elevated Mex67 levels observed in *Δhpr1* cells, which exhibit a nuclear mRNA export defect. Thus, an excessive Mex67 level in nuclear mRNPs, beyond that of wild-type cells, is likely detrimental for efficient nuclear mRNA export.

Taken together, we expand the current model of Mex67-Mtr2 function in nuclear mRNA export: In addition to the absence or malfunction of Mex67-Mtr2, an increased level of Mex67-Mtr2 in nuclear mRNPs can also impair nuclear mRNA export, underscoring the importance for its strict regulation. Notably, overexpression of Mex67-Mtr2 in wild-type cells increases the total Mex67 level in lysates but does not lead to an elevated level of Mex67—or of any other mRNP component—in nuclear mRNPs (Supplementary Fig. S4A and B). Consistently, these cells do not exhibit an mRNA export defect (Supplementary Fig. S4C). This suggests that the recruitment of Mex67-Mtr2 to mRNA and its level in nuclear mRNPs are tightly regulated. Supporting this idea, overexpression of Nab2 in wild-type cells increases Nab2 and Yra1 levels, but unlike in *Δhpr1* cells, does not elevate the Mex67 level in nuclear mRNPs (Supplementary Fig. S4D and E). Thus, higher Nab2 and Yra1 levels alone are insufficient to increase the Mex67 level in nuclear mRNPs. Consistently, overexpression of the adaptors Nab2, Yra1, or both in wild-type cells does not cause a nuclear mRNA export defect (Supplementary Fig. S4C). In summary, maintaining a wild-type level of Mex67-Mtr2 in nuclear mRNPs is essential for efficient nuclear mRNA export.

## Supplementary Material

gkag025_Supplemental_File

## Data Availability

The data supporting the findings of this study are available from the corresponding authors upon request.
