# Supplementary material for: An elevated level of the mRNA exporter Mex67-Mtr2 in nuclear mRNPs impairs nuclear mRNA export
Source: Nucleic Acids Res. 2026 Jan 23;54(3):gkag025. doi: 10.1093/nar/gkag025 (PMC12828223; doi:10.1093/nar/gkag025)
Supplement: gkag025_Supplemental_File [file gkag025_supplemental_file.pdf]

Supplementary Data

An elevated level of the mRNA exporter Mex67-Mtr2 in nuclear mRNPs impairs nuclear mRNA export

Nataliia Stefanyshena and Katja Sträßer

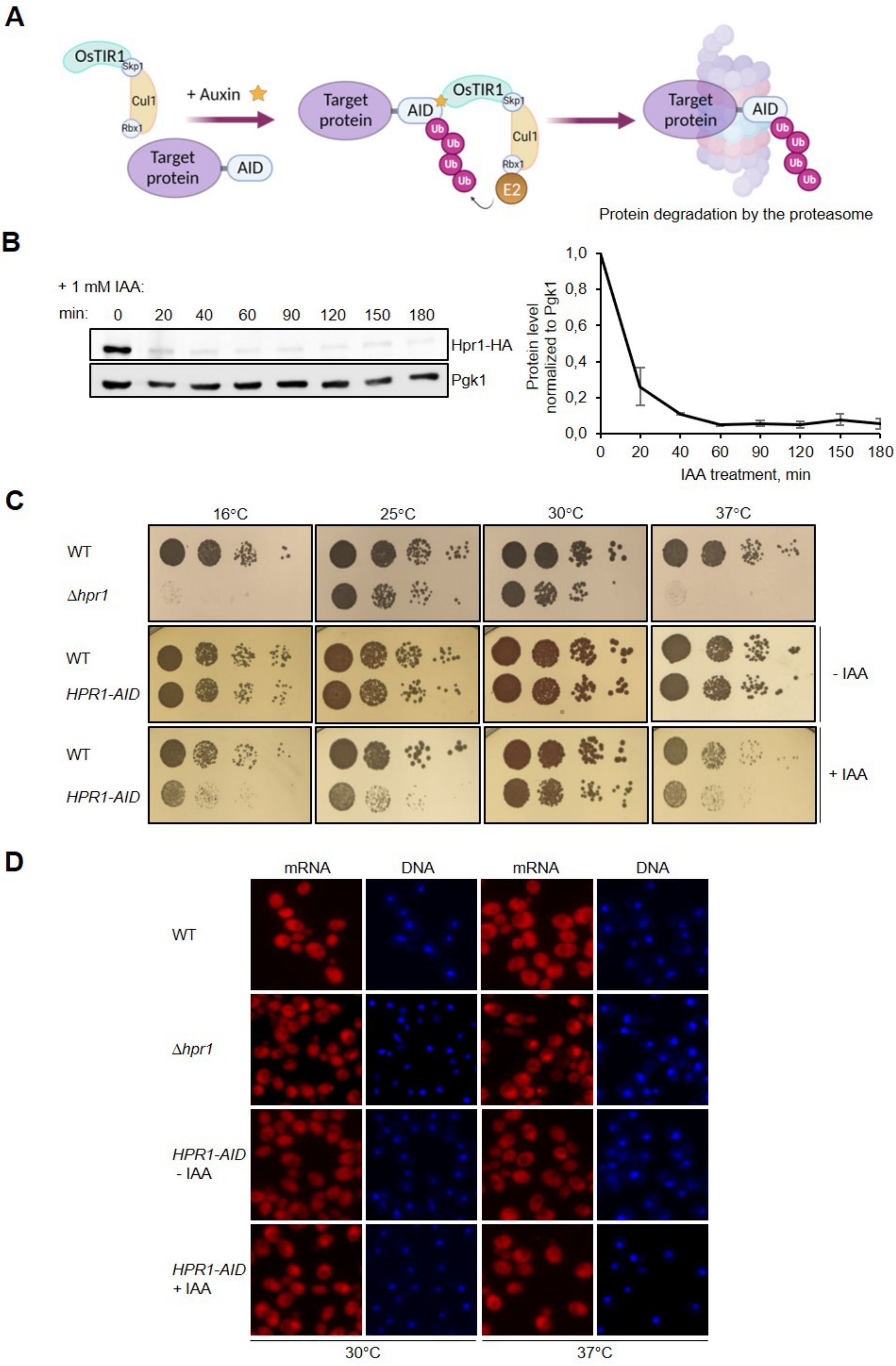
A
OsTIR1
Skp1
Cul1
Rbx1
Target protein
AID
+ Auxin
Ub
E2
Protein degradation by the proteasome
B
+ 1 mM IAA:
min: 0 20 40 60 90 120 150 180
Hpr1-HA
Pgk1
Protein level normalized to Pgk1
IAA treatment, min
C
16°C
25°C
30°C
37°C
WT
Δhpr1
HPR1-AID
- IAA
+ IAA
D
mRNA
DNA
HPR1-AID - IAA
HPR1-AID + IAA
30°C
37°C

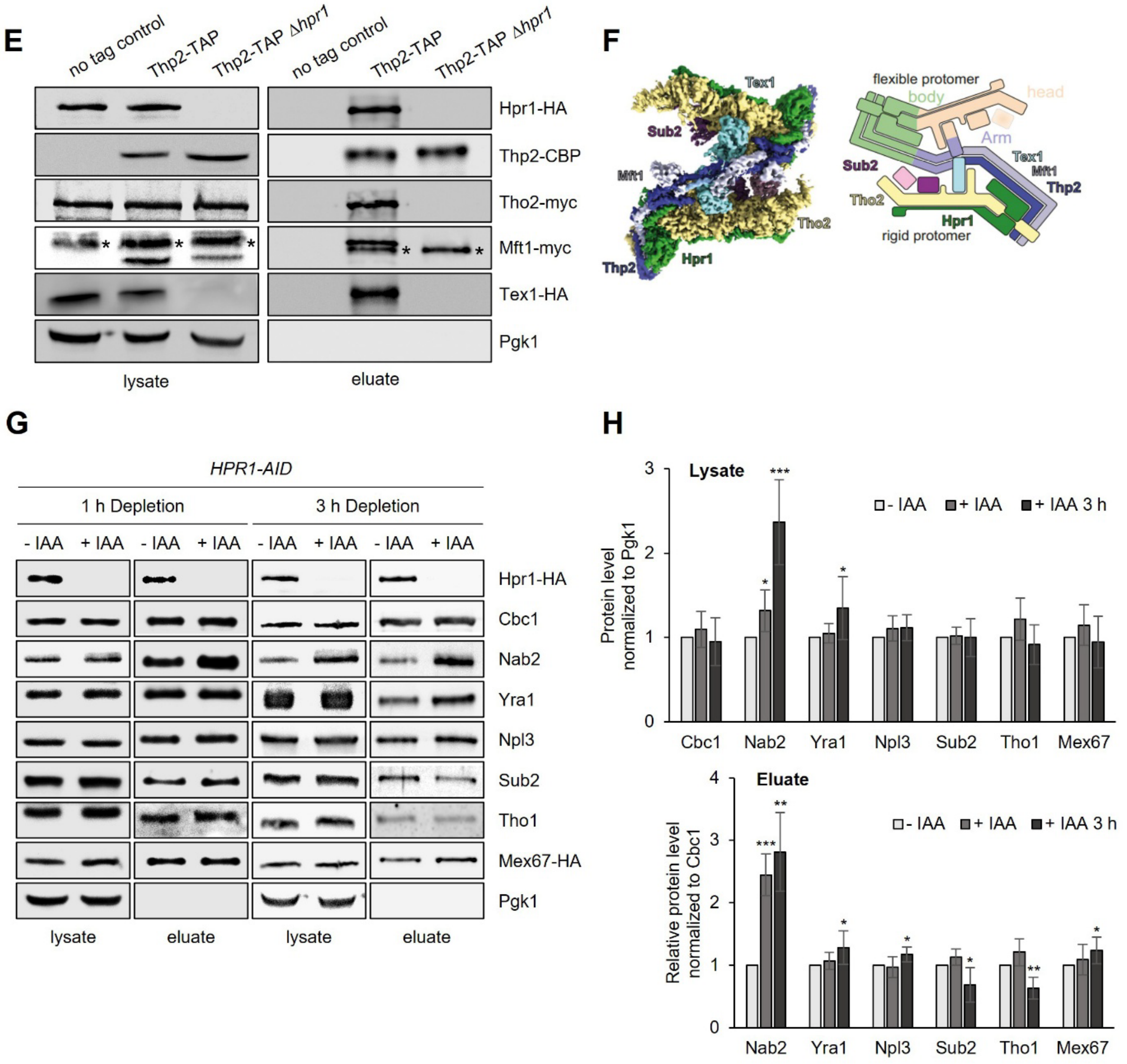

Supplementary Figure S1. Depletion of Hpr1 impairs cell growth and nuclear mRNA export and leads to an increased Nab2 level in nuclear mRNPs. (A) Scheme of the auxin-inducible degron (AID) system (modified from 1). Addition of auxin promotes the interaction between SCF E3 ligase complex subunit OsTIR1 and the AID tag of the target protein. This causes the recruitment of an E2 ligase that ubiquitinates the AID tag and thus targets the tagged protein for degradation by the proteasome. (B) Hpr1-AID is depleted after addition of auxin. Western blots of whole cell extracts after incubation of *HPR1-AID-HA* cells with 1 mM auxin (IAA) at different time points. Hpr1-AID-HA and Pgk1, which served as loading control, were detected with the respective antibodies. Hpr1 levels were quantified and normalized to Pgk1 levels. Data represents the mean ± standard deviation (SD). (C) Deletion or depletion of Hpr1 causes a growth defect. Ten-fold serial dilutions of WT and *Δhpr1* cells and of cells expressing Hpr1-AID were spotted onto YPD plates without (- IAA) or with 1 mM auxin (+ IAA), respectively, and grown at the indicated temperatures. (D) Deletion or depletion of Hpr1 causes a nuclear mRNA export defect. The localization of poly(A)+ RNA was determined at 30°C or after 1 h shift to 37°C of WT and *Δhpr1* cells and of *HPR1-AID* cells incubated without (- IAA) or with 1 mM auxin (+ IAA) for 1 h. Poly(A)+ RNA was visualized with oligo(dT)50-Cy3, DNA was stained with DAPI. (E) Deletion of *HPR1* leads to disintegration of the THO complex. Pull-down of the THO complex via Thp2-TAP in WT and *Δhpr1* cells. Western blots of lysates and TEV eluates after cleavage of the TAP tag, using antibodies against tagged components of the THO complex. Note, that the total amount of Tex1 decreases by deletion of *HPR1*. The position of

Mft1-myc is indicated by an asterisk. (F) Cryo-EM structure of the reconstituted THO-Sub2 complex (*left*) and its scheme (*right*) reproduced from (1). (G) Acute depletion of Hpr1 causes an elevated Nab2 level after one hour (+IAA) and elevated Nab2, Yra1 and Mex67 levels in nuclear mRNPs after three hours depletion (+IAA 3 h). Representative Western blots of lysates and TEV eluates after Cbc2-TAP purification from cells expressing Hpr1-AID without auxin treatment (- IAA) or with 1 mM auxin treatment for 1 h (+ IAA) or 3 h (+IAA 3 h) using antibodies against the indicated RBPs. (H) Quantification of the protein levels in lysates (*upper panel*) and TEV eluates (*lower panel*) normalized to Pgk1 or Cbc1 levels, respectively. Values for WT cells were set to 1. Data represents the mean ± SD from at least three independent experiments; *P < 0.05; **P < 0.01; ***P < 0.001.

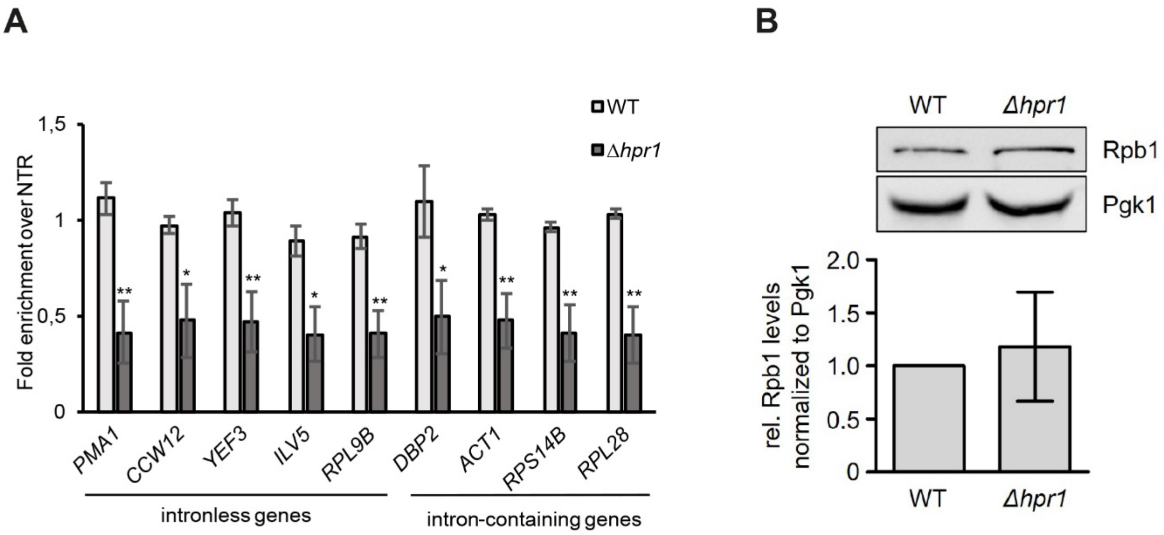

Supplementary Figure S2. RNAPII occupancy decreases in *Δhpr1* cells. (A) The occupancy of the RNAPII subunit Rpb1 at transcribed genes was assessed by chromatin immunoprecipitation (ChIP) using the antibody 8WG16, which recognizes the non-phosphorylated CTD. The occupancy was calculated as the enrichment of protein at the exemplary genes shown relative to its presence at a non-transcribed region (NTR, 174131–174200 on chr. V) and was set to 1 for the WT strain. Data represents the mean ± SD from at least three independent experiments; *P < 0.05; **P < 0.01. (B) Total RNAPII levels are unchanged in *Δhpr1* cells. Representative Western blots of lysates from WT and *Δhpr1* cells using antibodies against the indicated proteins (*upper panel*). Quantification of the protein levels in lysates normalized to Pgk1. Values for WT cells were set to 1. Data represents the mean ± SD from at least three independent experiments (*lower panel*).

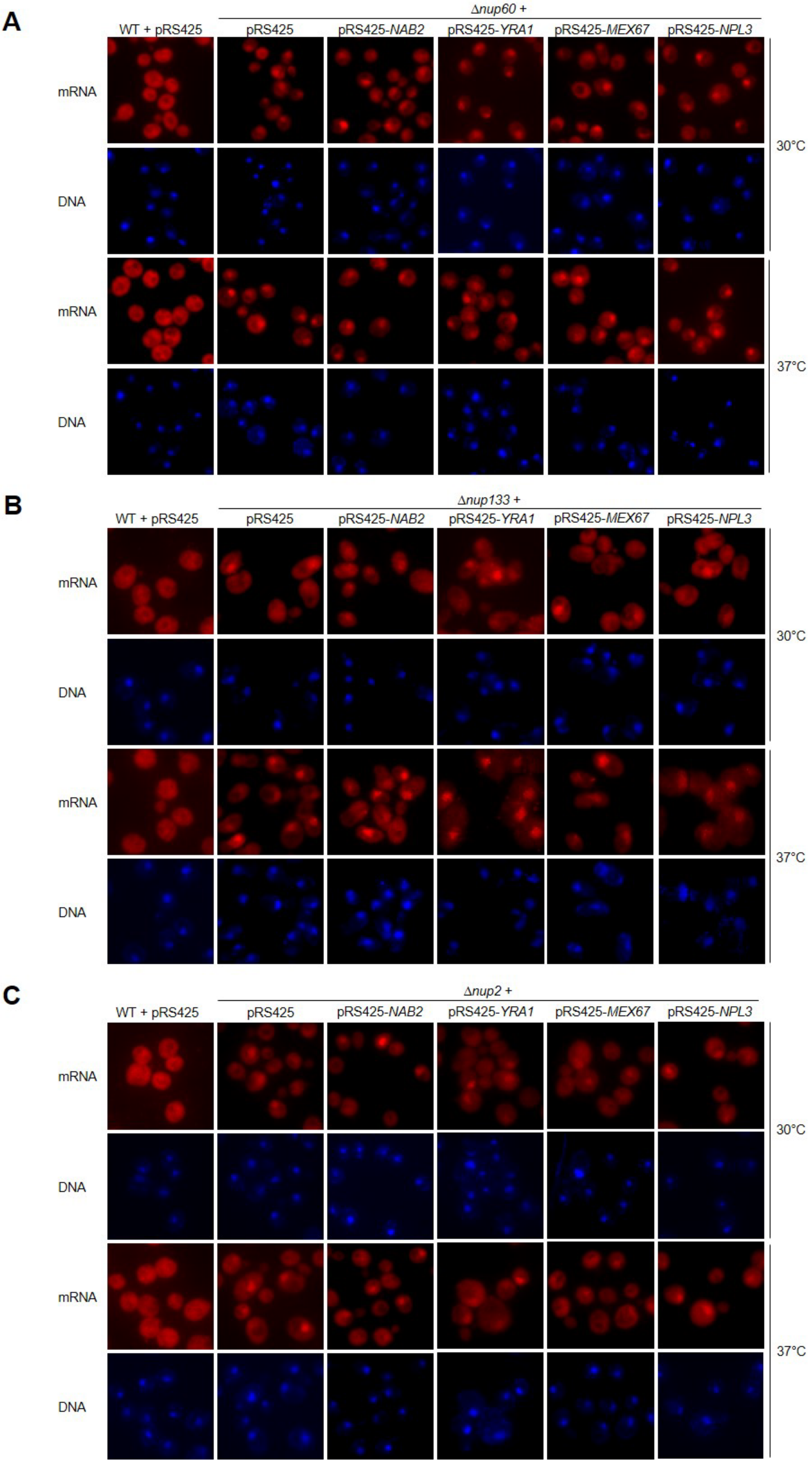
A
Δnup60 +
WT + pRS425
pRS425
pRS425-NAB2
pRS425-YRA1
pRS425-MEX67
pRS425-NPL3
mRNA
DNA
mRNA
DNA
30°C
37°C
B
Δnup133 +
WT + pRS425
pRS425
pRS425-NAB2
pRS425-YRA1
pRS425-MEX67
pRS425-NPL3
mRNA
DNA
mRNA
DNA
30°C
37°C
C
Δnup2 +
WT + pRS425
pRS425
pRS425-NAB2
pRS425-YRA1
pRS425-MEX67
pRS425-NPL3
mRNA
DNA
mRNA
DNA
30°C
37°C

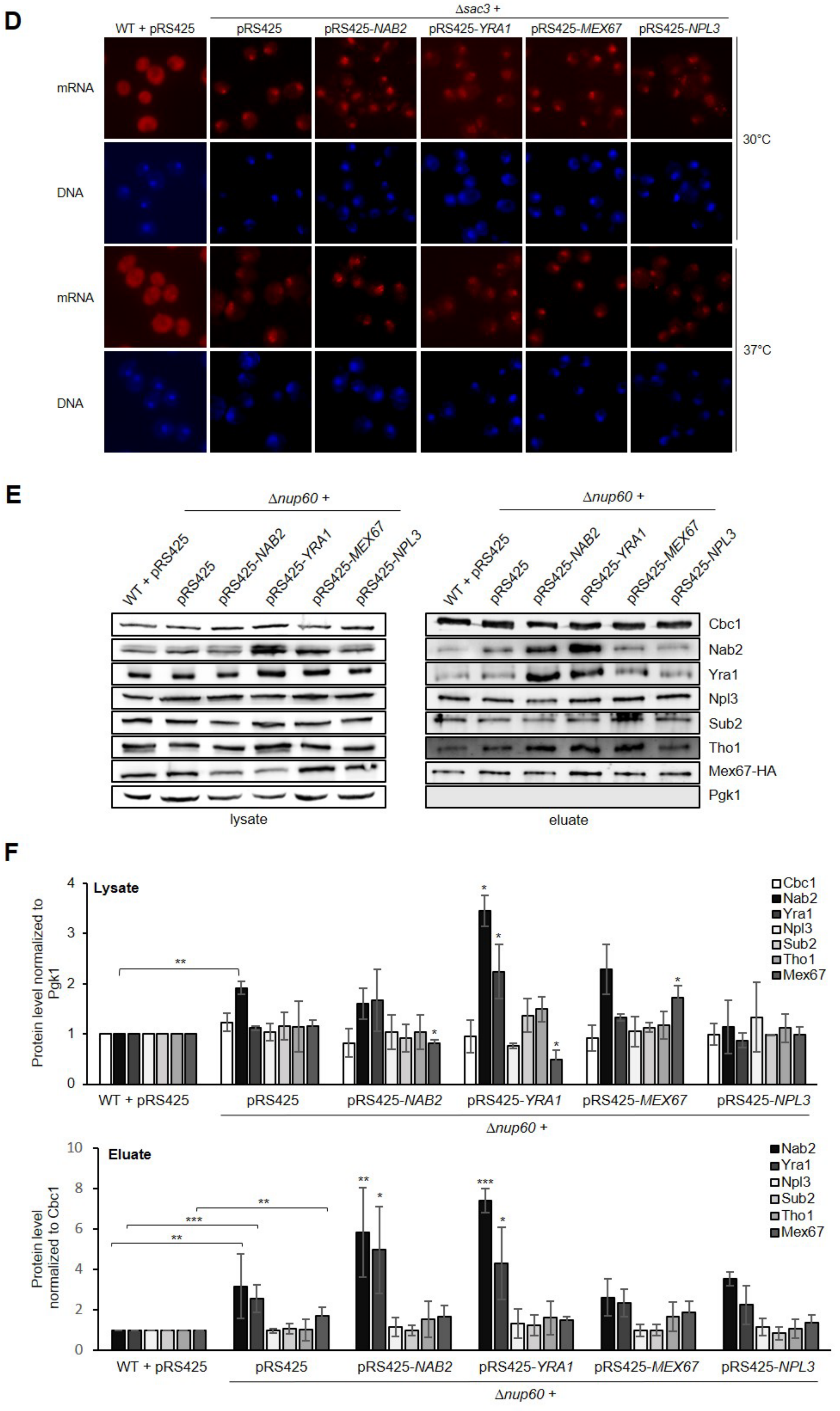

D
Δsac3 +
WT + pRS425
pRS425
pRS425-NAB2
pRS425-YRA1
pRS425-MEX67
pRS425-NPL3
mRNA
DNA
mRNA
DNA
30°C
37°C
E
Δnup60 +
Δnup60 +
WT + pRS425
pRS425
pRS425-NAB2
pRS425-YRA1
pRS425-MEX67
pRS425-NPL3
Cbc1
Nab2
Yra1
Npl3
Sub2
Tho1
Mex67-HA
Pgk1
lysate
eluate
F
Lysate
Protein level normalized to Pgk1
Cbc1
Nab2
Yra1
Npl3
Sub2
Tho1
Mex67
WT + pRS425
pRS425
pRS425-NAB2
pRS425-YRA1
pRS425-MEX67
pRS425-NPL3
Δnup60 +
Eluate
Protein level normalized to Cbc1
Nab2
Yra1
Npl3
Sub2
Tho1
Mex67
WT + pRS425
pRS425
pRS425-NAB2
pRS425-YRA1
pRS425-MEX67
pRS425-NPL3
Δnup60 +

Supplementary Figure S3. Overexpression of Nab2, Yra1, Mex67 or Npl3 does not suppress the nuclear mRNA export defect of *Δnup60*, *Δnup133, Δnup2* or *Δsac3* cells and does not decrease Mex67 levels in nuclear mRNPs to WT. (A-D) Deletion of *NUP60*, *NUP133*, *NUP2* or *SAC3* causes a nuclear mRNA export defect. Poly(A)+ RNA was visualized by fluorescence in situ hybridization (FISH) with an oligo(dT)50-Cy3 probe at 30°C or after 1 h shift to 37°C in the indicated strains. DNA was stained with DAPI. Representative images from three independent experiments are shown. (E and F) Overexpression of Nab2 or Yra1 in *Δnup60* cells leads to higher levels of Nab2 and Yra1 in nuclear mRNPs but does not decrease the level of Mex67 to WT levels. (E) Representative Western blots of lysates and TEV eluates after Cbc2-TAP purification in *Δnup60* cells and *Δnup60* cells overexpressing Nab2, Yra1, Mex67 or Npl3, using antibodies against the indicated RBPs. (F) Quantification of the protein levels in lysates and TEV eluates, normalized to the signal of Pgk1 or Cbc1, respectively. Protein levels of WT cells were set to 1. Asterisks with brackets represent the comparison between WT and *Δnup60* cells, asterisks without brackets indicate the comparison to *Δnup60* cells. *$P < 0.05$; **$P < 0.01$; ***$P < 0.001$.

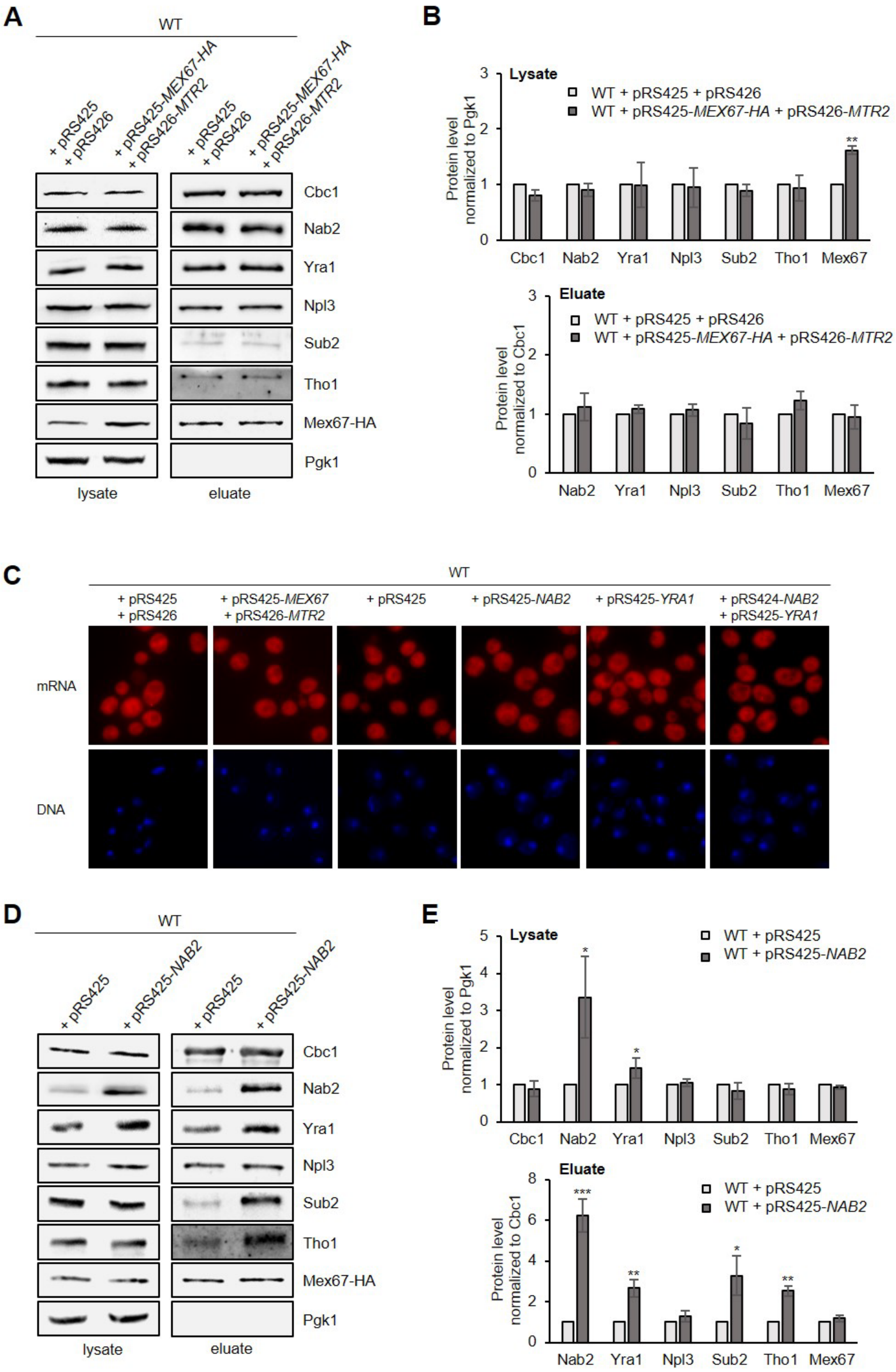
A
WT
+ pRS425 + pRS426
+ pRS425-MEX67-HA + pRS426-MTR2
Cbc1
Nab2
Yra1
Npl3
Sub2
Tho1
Mex67-HA
Pgk1
lysate
eluate
B
Lysate
WT + pRS425 + pRS426
WT + pRS425-MEX67-HA + pRS426-MTR2
Protein level normalized to Pgk1
Cbc1 Nab2 Yra1 Npl3 Sub2 Tho1 Mex67
Eluate
Protein level normalized to Cbc1
Nab2 Yra1 Npl3 Sub2 Tho1 Mex67
C
WT
+ pRS425 + pRS426
+ pRS425-MEX67 + pRS426-MTR2
+ pRS425
+ pRS425-NAB2
+ pRS425-YRA1
+ pRS424-NAB2 + pRS425-YRA1
mRNA
DNA
D
WT
+ pRS425
+ pRS425-NAB2
Cbc1
Nab2
Yra1
Npl3
Sub2
Tho1
Mex67-HA
Pgk1
lysate
eluate
E
Lysate
WT + pRS425
WT + pRS425-NAB2
Protein level normalized to Pgk1
Cbc1 Nab2 Yra1 Npl3 Sub2 Tho1 Mex67
Eluate
Protein level normalized to Cbc1
Nab2 Yra1 Npl3 Sub2 Tho1 Mex67

Supplementary Figure S4. Higher Nab2 and Yra1 levels do not result in an increased Mex67 level in nuclear mRNPs nor in a nuclear mRNA export defect. (A) Representative Western blots of lysates and TEV eluates of a Cbc2-TAP purification from WT cells or cells overexpressing Mex67-HA and Mtr2, using antibodies against the indicated RBPs. (B) Quantification of protein levels in lysates (upper panel) and TEV eluates (lower panel) normalized to levels of Pgk1 or Cbc1, respectively. Values for WT cells were set to 1. Data represents the mean ± SD of at least three independent experiments; **p <0.01. (C) Overexpression of Mex67 and Mtr2, Nab2, Yra1 or Nab2 and Yra1 does not cause a nuclear mRNA export defect. FISH of WT cells or cells overexpressing Mex67 and Mtr2, Nab2, Yra1 or Nab2 and Yra1 grown at 30°C. poly(A)+ RNA was detected using oligo(dT)50-Cy3 probes, DNA was stained with DAPI. Representative images from three independent experiments are shown. (D) Representative Western blots of lysates and TEV eluates of a Cbc2-TAP purification from WT cells or cells overexpressing Nab2, using antibodies against the indicated RBPs. (E) Quantification of the protein levels in lysates (upper panel) and TEV eluates (lower panel) as in (B). Data represents the mean ± SD of at least three independent experiments; *P < 0.05; **P < 0.01; ***P < 0.001.

Supplementary Table S1. Yeast strains

Strain	Genotype	Reference
W303	MATa, *ura3-1; trp1-1; his3-11;15, leu2-3,112; ade2-1; can1-100; GAL+*	(2)
Δ*hpr1*	MATa; *ura3-1; ade2-1; his3-11,5; trp1-1; leu2-3,112; can1-100; hpr1::HIS3*	R. Rothstein
CBC2-TAP MEX67-6xHA	MATa; *ura3-1; trp1-1; his3-11,15; leu2-3,112; ade2-1; can1-100; GAL+; CBC2-CBP-TEV-protA::TRP1; MEX67-HA::KanMX4*	This study
Δ*hpr1 CBC2-TAP MEX67-6xHA*	MATa; *ura3-1; trp1-1; his3-11,15; leu2-3,112; ade2-1; can1-100; GAL+; hpr1::HIS3; CBC2-CBP-TEV-protA::TRP1; MEX67-HA::KanMX4*	This study
NAB2-TAP	MATa; *ura3-1; trp1-1; his3-11,15; leu2-3,112; ade2-1; can1-100; GAL+; NAB2-CBP-TEV-protA::TRP1*	This study
Δ*hpr1 NAB2-TAP*	MATa; *ura3-1; trp1-1; his3-11,15; leu2-3,112; ade2-1; can1-100; GAL+; NAB2-CBP-TEV-protA::TRP1; hpr1::HIS3*	This study
YRA1-TAP	MATa; *ura3-1; trp1-1; his3-11,15; leu2-3,112; ade2-1; can1-100; GAL+; YRA1-CBP-TEV-protA::TRP1*	This study
Δ*hpr1 YRA1-TAP*	MATa; *ura3-1; trp1-1; his3-11,15; leu2-3,112; ade2-1; can1-100; GAL+; Tho2-9myc::KanMX4; hpr1::HIS3; YRA1-CBP-TEV-protA::TRP1*	This study
MEX67-TAP	MATa; *ura3-1; trp1-1; his3-11,15; leu2-3,112; ade2-1; can1-100; GAL+; MEX67-CBP-TEV-protA::TRP1*	This study
Δ*hpr1 MEX67-TAP*	MATa; *ura3-1; trp1-1; his3-11,15; leu2-3,112; ade2-1; can1-100; GAL+; Tho2-9myc::KanMX4; hpr1::HIS3; MEX67-CBP-TEV-protA::TRP1*	This study
Δ*nup60*	MATa; *ura3-1; trp1-1; his3-11,15; leu2-3,112; ade2-1; can1-100; GAL+; nup60::HIS3*	This study
Δ*nup133*	MATa; *ura3-1; trp1-1; his3-11,15; leu2-3,112; ade2-1; can1-100; GAL+; nup133::HIS3*	This study
Δ*nup2*	MATa; *ura3-1; trp1-1; his3-11,15; leu2-3,112; ade2-1; can1-100; GAL+; nup2::HIS3*	This study
Δ*nup60 CBC2-TAP MEX67-6xHA*	MATa; *ura3-1; trp1-1; his3-11,15; leu2-3,112; ade2-1; can1-100; GAL+; nup60::HIS3; CBC2-CBP-TEV-protA::TRP1; MEX67-HA::KanMX4*	This study
Δ*nup133 CBC2-TAP MEX67-6xHA*	MATa; *ura3-1; trp1-1; his3-11,15; leu2-3,112; ade2-1; can1-100; GAL+; nup133::HIS3; CBC2-CBP-TEV-protA::TRP1; MEX67-HA::KanMX4*	This study
Δ*nup2 CBC2-TAP MEX67-6xHA*	MATa; *ura3-1; trp1-1; his3-11,15; leu2-3,112; ade2-1; can1-100; GAL+; nup2::HIS3; CBC2-CBP-TEV-protA::TRP1; MEX67-HA::KanMX4*	This study

RS453	MATa, *ade2-1; his3-11,15; ura3-52; leu2-3,112; trp1-1; can1-100; GAL+*	(3)
Δhpr1	MATa; *ade2-1; his3-11,15; ura3-52; leu2-3,112; trp1-1; can1-100; GAL+; hpr1::KanMX4*	This study
Δsac3	MATa; *ade2-1; his3-11,15; ura3-52; leu2-3,112; trp1-1; can1-100; GAL+; sac3::HIS3*	This study
Δhpr1 CBC2-TAP MEX67-6xHA	MATa; *ade2-1; his3-11,15; ura3-52; leu2-3,112; trp1-1; can1-100; GAL+; hpr1::KanMX4; CBC2-CBP-TEV-protA::TRP1; MEX67-HA::HIS3*	This study
Δsac3 CBC2-TAP MEX67-6xHA	MATa; *ade2-1; his3-11,15; ura3-52: leu2-3,112; trp1-1; can1-100; GAL+; sac3::HIS3; CBC2-CBP-TEV-protA::TRP1; MEX67-HA::KanMX4*	This study
SUB2 shuffle *CBC2-TAP MEX67-6xHA*	MATa; *ura3-1; trp1-1; his3-11,15; leu2-3,112; ade2-1; can1-100; GAL+; CBC2-CBP-TEV-protA::TRP1; MEX67-HA::HIS3; sub2::KanMX4*; pRS316-*SUB2*	This study
osTIR HPR1-AID CBC2-TAP MEX67-6xHA	MATa; *ura3-1; trp1-1; his3-11,15; leu2-3,112; ade2-1; can1-100; GAL+; URA3::osTIR HPR1-AID-6HA::Hyg; CBC2-CBP-TEV-protA::TRP1; MEX67-6HA::KanMX4*	This study
THO2-9myc TEX1-6HA MFT1-3myc HPR1-6HA THP2-TAP	MATa; *ura3-1; trp1-1; his3-11,15; leu2-3,112; ade2-1; can1-100; GAL+; THO2-9myc::KanMX4; TEX1-6HA::LEU; MFT1-3myc::TRP1; HPR1-6HA::HIS3; THP2-TAP::URA3*	This study
Δhpr1 THO2-9myc TEX1-6HA MFT1-3myc HPR1-6HA THP2-TAP	MATa; *ura3-1; trp1-1; his3-11,15; leu2-3,112; ade2-1; can1-100; GAL+; THO2-9myc::KanMX4; hpr1::HIS3; TEX1-6HA::LEU; MFT1-3myc::TRP1; THP2-TAP::URA3*	This study

Supplementary Table S2. Plasmids

Name	Description	Reference
pBS1479	For genomic C-terminal TAP-tagging (CBP-TEV-2x protein A), TRP1-KL	(4)
pYM14	For genomic C-terminal 6xHA-tagging, KanMX4	Euroscarf
pRS425	High copy plasmid for overexpression, *LEU2* marker	(5)
pRS425-*NAB2*	ORF + 500 bp of 5’ and 500 bp of 3’ UTR of *NAB2* was cloned into pRS425	This study
pRS425-*YRA1*	ORF + 500 bp of 5’ and 500 bp of 3’ UTR of *YRA1* was cloned into pRS425	This study
pRS425-*MEX67*	ORF + 500 bp of 5’ and 500 bp of 3’ UTR of *MEX67* was cloned into pRS425	This study
pRS425-*MEX67-6HA*	ORF of *MEX67* was C-terminal tagged with 6HA; + 500 bp of 5’ and 500 bp of 3’ UTR	This study

pRS425-*NPL3*	ORF + 500 bp of 5' and 500 bp of 3' UTR of *NPL3* was cloned into pRS425	(6)
pNOP1-*SUB2*	*NOP1* promoter, *SUB2* ORF + 500 bp of 5' and 500 bp of 3' UTR, *LEU2* marker	This study
pGAL1-*SUB2*	*GAL1* promoter, *SUB2* ORF + 500 bp of 5' and 500 bp of 3' UTR, *LEU2* marker	This study

Supplementary Table S3. Primers

Primers used for cloning:

**Name**	**Sequence (5'→3')**
pRS425-*NAB2* vector fwr	AAAATTATTTAATGGTTGATGAATTCCTGCAGCCCGG
pRS425-*NAB2* vector rev	TGTAATGAACCACACGATCGGATATCAAGCTTATCGATACCGTCG
pRS425-*NAB2* fragment fwr	GTATCGATAAGCTTGATATCCGATCGTGTGGTTCATTACA
pRS425-*NAB2* fragment rev	CCCCCGGGCTGCAGGAATTCATCAACCATTAAATAATTTTGTACACTTATAATAACAC
pRS425-*YRA1* vector fwr	TCATAATAGTTTTTTGAATTCCTGCAGCCCGGG
pRS425-*YRA1* vector rev	CGGGGAAATCCAATTGATATCAAGCTTATCGATACCGTCGAC
pRS425-*YRA1* fragment fwr	GATAAGCTTGATATCAATTGGATTTCCCCGAACAGC
pRS425-*YRA1* fragment rev	GGGCTGCAGGAATTCAAAAAACTATTATGATAACCTTGCTCAAGTCAAAC
pRS425-*MEX67* vector fwr	CTTGGACCGTAAAACTGCCTGCAGCCCGGGGGATCCACT
pRS425-*MEX67* vector rev	AAAATTTGGAGGACGAATTCGATATCAAGCTTATCGATACCGT
pRS425-*MEX67* fragment fwr	GCTTGATATCGAATTCGTCCTCCAAATTTTGCACCTC
pRS425-*MEX67* fragment rev	GATCCCCCGGGCTGCAGGCAGTTTTACGGTCCAAGCGG
pRS425-*MEX67-6HA* vector fwr	CTCGAATTCATCGATTAATGATATTGTTCCCTGTTTCAGCCG
pRS425-*MEX67-6HA* vector rev	GACCTGCAGCGTACGGAACTGCACAAATGCTTCTCTAGG
pRS425-*MEX67-6HA* fragment fwr	TAAACTGTATATTTTTTGTGATACTGTGCGGCTGAAACAGGGAACAATATCATTAATCGATGAATTCGAGCTCG
pRS425-*MEX67-6HA* fragment rev	AAAGGGTTTTCAGAGTAGCATGAATGGCATCCCTAGAGAAGCATTTGTGCAGTTCCGTACGCTGCAGGTCGAC

Primers used for qPCRs in ChIP and RIP experiments:

**Name**	**Sequence (5'→3')**
NTR fwd (7)	TGCGTACAAAAAGTGTCAAGAGATT
NTR rev (7)	ATGCGCAAGAAGGTGCCTAT
PMA1 3' fwd	CAGAGCTGCTGGTCCATTCTG
PMA1 3' rev	GAAGACGGCACCAGCCAAT
CCW12 3' fwd	TGAAGCTCCAAAGAACACCACC
CCW12 3' rev	AGCAGCAGCACCAGTGTAAG
YEF3 3' fwd	TCTGGTCACAACTGGGTTAGTG
YEF3 3' rev	GCAATCTTGTTACCCATAGCATCGA
ILV5 3' fwd	TGGTACCCAATCTTCAAGAATGC
ILV5 3' rev	ACCGTTCTTGGTAGATTCGTACA
RPL9B 3' fwd	AGGACGAAATCGTCTTATCTGGT
RPL9B 3' rev	CAGATTTGTTGCAAGTCAGCGG
DBP2 3' fwd	CTTCACCGAACAAAACAAAGGTT
DBP2 3' rev	TCGGGAGGAATATTTTGATTAGCT
ACT1 3' fwd	ATCATGAAGTGTGATGTCGATGTC
ACT1 3' rev	ATGGTGGTACCACCGGACATAA
RPS14B 3' fwd	AAGACCCCAGGACCAGGTG
RPS14B 3' rev	GATACGGCCAATCCTCAAACCAG
PRL28 3' fwd	TGGAAGCCAGTCTTGAACTTGG
PRL28 3' rev	TTGGTCTCTCTTGTCTTCTGGGA

REFERENCES

1. Schuller, S.K., Schuller, J.M., Prabu, J.R., Baumgartner, M., Bonneau, F., Basquin, J. and Conti, E. (2020) Structural insights into the nucleic acid remodeling mechanisms of the yeast THO-Sub2 complex. *eLife*, **9**.
2. Thomas, B.J. and Rothstein, R. (1989) Elevated recombination rates in transcriptionally active DNA. *Cell*, **56**, 619-630.
3. Strasser, K. and Hurt, E. (2000) Yra1p, a conserved nuclear RNA-binding protein, interacts directly with Mex67p and is required for mRNA export. *Embo J*, **19**, 410-420.
4. Puig, O., Caspary, F., Rigaut, G., Rutz, B., Bouveret, E., Bragado-Nilsson, E., Wilm, M. and Seraphin, B. (2001) The tandem affinity purification (TAP) method: a general procedure of protein complex purification. *Methods*, **24**, 218-229.
5. Sikorski, R.S. and Hieter, P. (1989) A system of shuttle vectors and yeast host strains designed for efficient manipulation of DNA in Saccharomyces cerevisiae. *Genetics*, **122**, 19-27.
6. Keil, P., Wulf, A., Kachariya, N., Reuscher, S., Huhn, K., Silbern, I., Altmuller, J., Keller, M., Stehle, R., Zarnack, K. *et al.* (2023) Npl3 functions in mRNP assembly by recruitment of mRNP components to the transcription site and their transfer onto the mRNA. *Nucleic Acids Res*, **51**, 831-851.
7. Mayer, A., Lidschreiber, M., Siebert, M., Leike, K., Soding, J. and Cramer, P. (2010) Uniform transitions of the general RNA polymerase II transcription complex. *Nature structural & molecular biology*, **17**, 1272-1278.
